# Supplementary figures and images for: Exploring a pico-well based scRNA-seq method (HIVE) for simplified processing of equine bronchoalveolar lavage cells
Source: PLoS One. 2025 Jan 24;20(1):e0317343. doi: 10.1371/journal.pone.0317343 (PMC11760581; doi:10.1371/journal.pone.0317343)

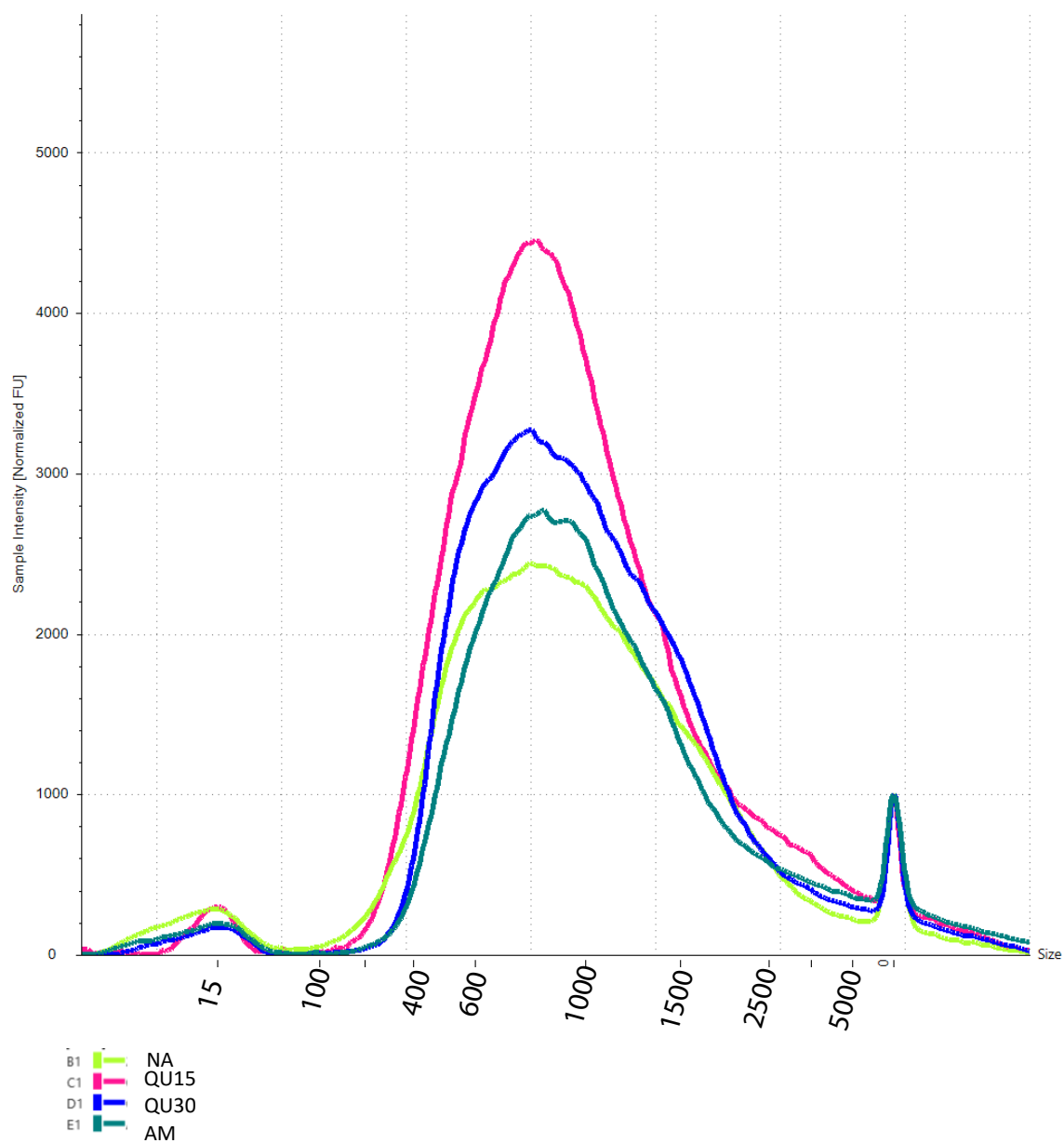

**S1 Figure.** Example of TapeStation size profiles for HIVE libraries

Supplement: S1 Fig — (PDF) [file pone.0317343.s001.pdf]
