# Supplementary material for: Exploring a pico-well based scRNA-seq method (HIVE) for simplified processing of equine bronchoalveolar lavage cells
Source: PLoS One. 2025 Jan 24;20(1):e0317343. doi: 10.1371/journal.pone.0317343 (PMC11760581; doi:10.1371/journal.pone.0317343)
Supplement: S2 Fig — (PDF) [file pone.0317343.s002.pdf]

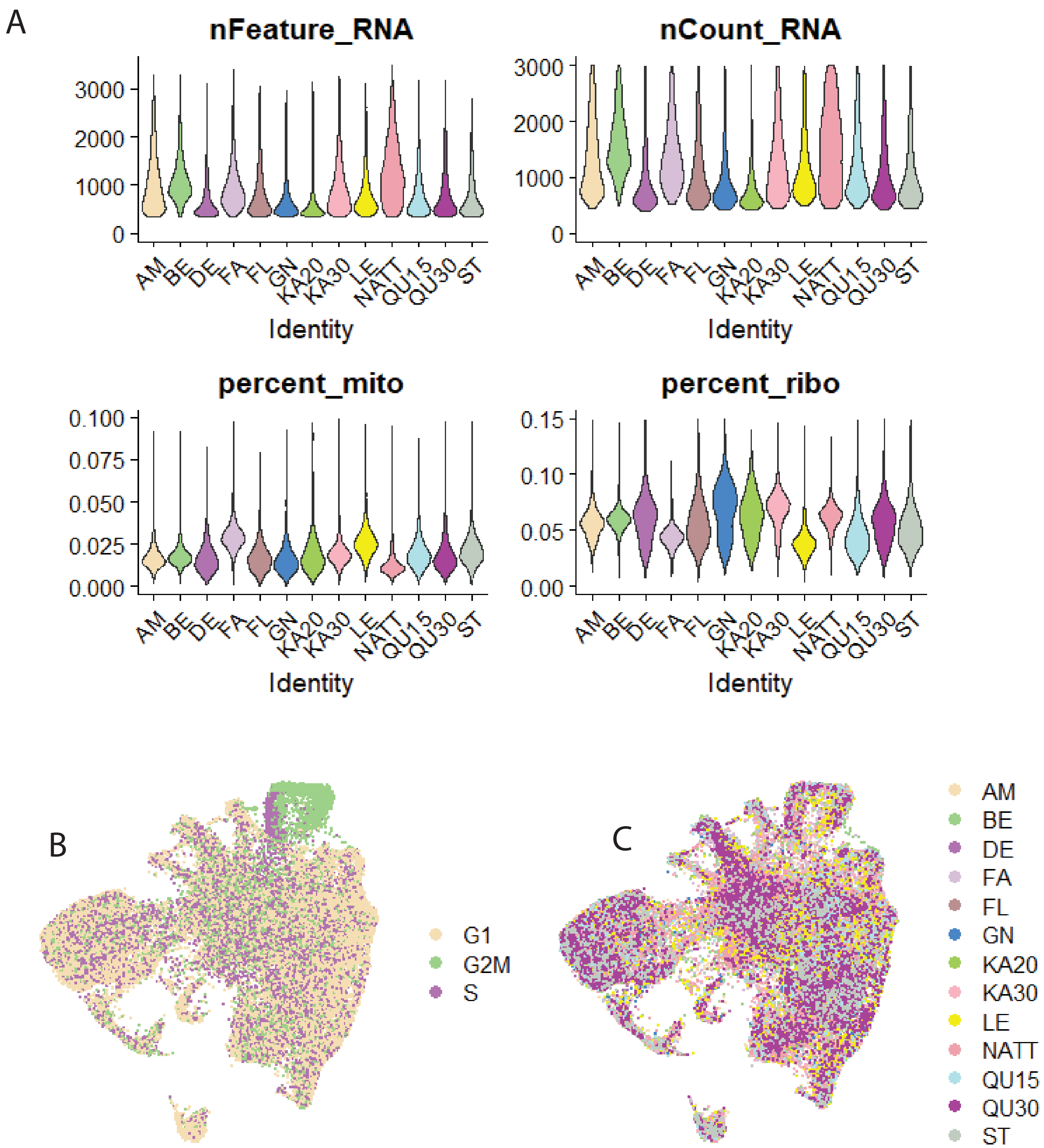

S2 Figure.
A. Violin plots showing QC metrics for 13 HIVE libraries. Genes (nFeatures)/cell. UMIs (nCounts)/cell. Percent mitochondrial and ribosomal counts/cell.
B. UMAP plot showing cells colored by cell cycle scores.
C. UMAP plot showing cells color by sample identity.
