# Supplementary material for: Exploring a pico-well based scRNA-seq method (HIVE) for simplified processing of equine bronchoalveolar lavage cells
Source: PLoS One. 2025 Jan 24;20(1):e0317343. doi: 10.1371/journal.pone.0317343 (PMC11760581; doi:10.1371/journal.pone.0317343)
Supplement: S3 Fig — (PDF) [file pone.0317343.s003.pdf]

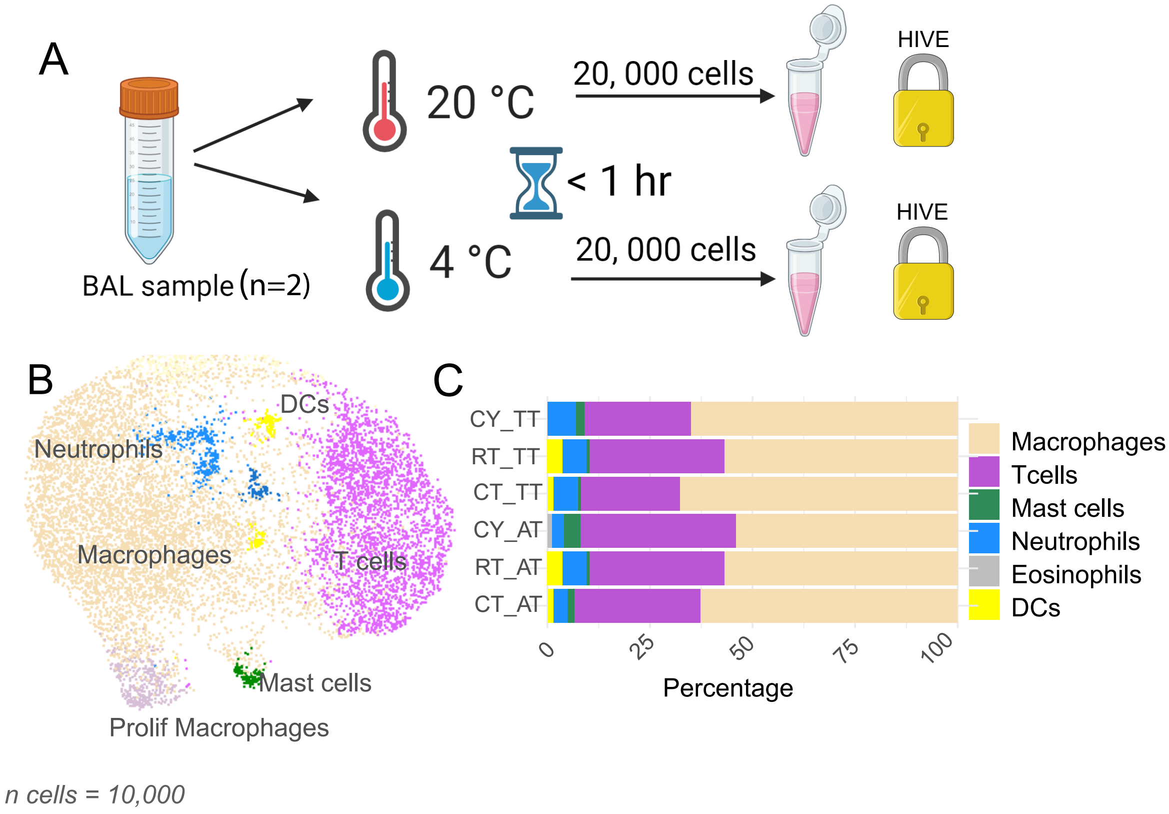

S3. Figure Cell type distribution in HIVE libraries prepared with alternative sample handling conditions.

A) Four HIVE libraries were prepared from two BAL samples (IDs: AT & TT) using alternative sample buffers and different sample handling temperatures. Each BAL sample was promptly split into two aliquots and kept either cold or at ambient temperature. Cells were then diluted in cold or ambient temperature cell media buffer (RPMI) supplemented with 5% FBS and RNase inhibitor, directly prior to loading the collectors, which took place in less than one hour after the BAL sampling procedure (as opposed to 2-4 hours in the initial experiments).

B) Clustering of integrated data integrated from the four libraries and labelling of the major cell types in BAL.

C) Cell type compositions are represented in bar plots and compared to cytology. Although the proportions of macrophages, T cells, and neutrophils appear similar to those in cytology, this interpretation should be made with caution due to the low number of replicates, which precludes statistical testing. Mast cell numbers were still lower in the HIVE data, and eosinophils were not detected at all. CY = cytology, RT = HIVE room temperature, CT = HIVE cold temperature.
